# Southern Association of Railway Surgeons

**Published:** 1898-06

**Authors:** 


### SOUTHERN ASSOCIATION OF RAILWAY SURGEONS.

A movement is now on foot for the formation of a "Southern Association of Railway Surgeons." This, we believe, is a much-needed organization. The movement is being put forward by the members of the Association of the Southern Railway Surgeons, and especially by its President, Dr. C. M. Drake. The annual meeting of this latter association is to be held at Old Point Comfort June 21 and 22. An invitation has been extended to the surgeons connected with other Southern railways, and it is hoped that such an organization will be effected. The meeting at Old Point Comfort promises to be one of great interest, and a most excellent program is already assured.

The following circular has been sent out by Dr. Drake:

"I beg to inclose herewith an announcement of the third annual meeting of the Association of Surgeons of the Southern Railway Company, at the Hygeia Hotel, Old Point Comfort, Va., June 21 and 22, 1898, and to extend an invitation to you to be present and to participate in the proceedings.

"At the same time and place it is proposed to hold a conference of chief surgeons or other medical representatives of the principal railroads operating in Virginia, West Virginia, North and South Carolina, Georgia, Florida, Alabama, Mississippi, Louisiana, Texas, Arkansas, Tennessee and Kentucky, for the purpose of determining the advisability of forming an organization to be known as the "Southern Association of Railway Surgeons," or some other equally comprehensive name.

"There are many good reasons for such an organization. The National Association of Railway Surgeons is too large to do effective work; its places of meeting are often too far removed for the annual attendance of the majority of Southern members, and of late many of the large systems have tacitly discouraged the attendance of their surgeons, and have declined to issue or request transportation on account of its meetings.

"It is hoped that you will attend the Old Point meeting."

Invitations have been extended to the surgeons of the Norfolk and Western Railroad, Chesapeake and Ohio Railroad, Atlantic Coast Line, Seaboard Air Line, New York, Philadelphia and Norfolk Railroad, Atlantic and Danville Railroad, and Norfolk and Southern Railroad to attend the meeting and participate in the deliberations of the Association, and doubtless these roads will be well represented.

The following distinguished surgeons have promised to be present and to deliver addresses on subjects pertaining to railway surgery: Dr. John A. Wyeth, New York; Drs. W. W. Keen and Joseph Price, Philadelphia; Dr. Hunter McGuire, Richmond; Dr. Walter Wyman, Surgeon-General, U. S. Marine Hospital Service; Dr. Joseph Ransahoff, Cincinnati; Dr. Willis F. Westmoreland, Atlanta, and Dr. J. J. Kinyoun, Past Assistant Surgeon, U. S. Marine Hospital Service.

Altogether, a most interesting meeting is promised. The place and the occasion will be well worth a visit from all railway surgeons.

